# Simulation of enteric pathogen concentrations in locally-collected greywater and wastewater for microbial risk assessments

**DOI:** 10.1016/j.mran.2016.11.001

**Published:** 2017-04

**Authors:** Michael A. Jahne, Mary E. Schoen, Jay L. Garland, Nicholas J. Ashbolt

**Affiliations:** a U.S. Environmental Protection Agency, 26 W. Martin Luther King Dr., Cincinnati OH 45268, United States; b Soller Environmental, 3022 King St., Berkeley, CA 94703, United States; c University of Alberta, Rm 3-57D South Academic Building, Edmonton, AB T6G 2G7, Canada

**Keywords:** Greywater, Wastewater, Decentralized systems, Water reuse, Waterborne pathogens, Microbial risk assessment

## Abstract

As decentralized water reuse continues to gain popularity, risk-based treatment guidance is increasingly sought for the protection of public health. However, effort s to evaluate pathogen risks and log-reduction requirements have been hindered by an incomplete understanding of pathogen occurrence and densities in locally-collected wastewaters (*i.e.*, from decentralized collection systems). Of particular interest is the potentially high enteric pathogen concentration in small systems with an active infected excreter, but generally lower frequency of pathogen occurrences in smaller systems compared to those with several hundred contributors. Such variability, coupled with low concentrations in many source streams (*e.g.*, sink, shower/bath, and laundry waters), has limited direct measurement of pathogens. This study presents an approach to modeling pathogen concentrations in variously sized greywater and combined wastewater collection systems based on epidemiological pathogen incidence rates, user population size, and fecal loadings to various residential wastewater sources. Pathogen infections were modeled within various population sizes (5-, 100-, and 1,000-person) for seven reference pathogens (viruses: adenoviruses, *Norovirus*, and *Rotavirus*; bacteria: *Campylobacter* and *Salmonella* spp.; and protozoa: *Cryptosporidium* and *Giardia* spp.) on each day of 10,000 possible years, accounting for intermittent infection and overlap of infection periods within the population. Fecal contamination of fresh greywaters from bathroom sinks, showers/baths, and laundry, as well as combined greywater and local combined wastewater (*i.e.*, including toilets), was modeled based on reported fecal indicators in the various sources. Simulated daily infections and models of fecal contamination were coupled with pathogen shedding characteristics to generate distributions of pathogen densities in the various waters. The predicted frequency of pathogen occurrences in local wastewaters was generally low due to low infection incidence within small cohort groups, but increased with collection scale (population size) and infection incidence rate (*e.g.*, *Norovirus)*. When pathogens did occur, a decrease in concentrations from 5- to 100- and from 100- to 1,000-person systems was observed; nonetheless, overall mean concentrations (*i.e.*, including non-occurrences) remained the same due to the increased number of occurrences. This highlights value of the model for characterizing scaling effects over averaging methods, which overestimate the frequency of pathogen occurrence in small systems while underestimating concentration peaks that likely drive risk periods. Results of this work will inform development of risk-based pathogen reduction requirements for decentralized water reuse.

## Introduction

Limited water resources and the rising awareness of conservation potential has led to an increased interest in water reuse. Onsite or local collection, treatment, and reuse of household wastewater or greywater offers the practical opportunity to provide water savings while minimizing the cost and liability of centralized infrastructure, particularly when coupled with energy recovery (Xue et al., 2015). However, compared to municipal sewage these waters experience large variations in quality due to lack of wastewater dilution, sporadic pathogen occurrences, and variability in user behavior, and their pathogen content is poorly characterized (O’Toole et al., 2014; Schoen and Garland, 2015). This has precluded the development of pathogen-based treatment standards for decentralized water reuse, resulting in existing standards that specify treatment parameters that are routine to measure yet lacking a demonstrated relationship to pathogen risk (NSF/ANSI, 2012). Indeed, a recent assessment of greywater reuse potential by the National Academy of Sciences concluded that household or multi-residential scale systems can offer cost-effective reductions in water demand, but that expansion is hindered by lack of risk-based reuse guidelines. Better understanding of pathogen occurrence and fate in these systems is necessary for determination of fit-for-purpose treatment requirements (NAS, 2016).

Attempts to measure enteric pathogens directly in greywater have been largely ineffective, often experiencing non-detects (Benami et al., 2015; Christova-Boal et al., 1996; Winward et al., 2008) (Table 1). To a large extent, these non-detects may occur due to intermittent infection incidence among smaller population sizes (*e.g.*, single households, apartment buildings/subdivisions, or blocks/districts as opposed to entire cities). Conversely, as the population size increases, wastewater dilution effects result in a more stabilized low pathogen concentration; with the large contributing population of municipal wastewater, *Norovirus* concentrations during outbreak conditions remain comparable to those that are routinely observed (Hellmér et al., 2014; Pouillot et al., 2015). Given quantitative limits of detection for any method, this interplay creates difficulty in measurement and interpretation of non-detect results; pathogens may indeed be present but below detection limits, or present at other times not captured by the sampling campaign. The few studies containing positive detections (Table 1) have either been non-quantitative (O’Toole et al., 2012) or too limited (system-specific or insufficiently reported) for broad applicability (Birks et al., 2004; Birks and Hills, 2007; Kim et al., 2009).

Others have attempted to estimate pathogen content based on the ratio of pathogens to fecal indicators in municipal wastewater (Deere et al., 2006; NRMMC-EPHC-AHMC, 2006; Maimon et al., 2010), but poor correlations and scaling effects limit the ability of such methods to accurately characterize onsite waters (O’Toole et al., 2014). An alternative approach is the use of indicators to determine fecal contamination of the water and epidemiological data to estimate pathogens shed in that feces (Barker et al., 2013a; Ottoson and Stenström, 2003; Schoen et al., 2014; Fane et al., 2002). These models have often determined pathogen concentrations using the number of infections averaged over an annual (Ottoson and Stenström, 2003), seasonal (Mok et al., 2014), or monthly basis (Barker et al., 2013a). However, this simplification results in a small number of fractional infections on each day, an impossible description of actual conditions. Implicitly, such models assume that pathogens are always present but in low concentrations, and neglect scaling effects as infections are averaged over the same population in which dilution occurs. In order for (low) annual infection incidence rates to be correct, the requirement of whole numbers of infections implies that in small populations there are often days in which no infection occurs. No models have been developed for a comprehensive suite of fecal pathogens and source water types while accounting for scaling effects.

The objective of this work was to simulate enteric pathogen occurrence and concentrations in various local wastewaters (source-separated greywaters, combined greywater, and total domestic wastewater) as a function of collection scale (population size). This was accomplished by coupling literature review of fecal contamination of fresh greywater from bathroom sinks, showers/baths, and laundry, as well as of local wastewater from all sources including toilets, with a model of infection occurrence in small populations and reported pathogen shedding characteristics (durations and fecal densities). These results are intended to support the development of risk-based treatment guidance for the safe reuse of human-impacted wastewaters (Schoen et al., 2016).

## Methods

Enteric pathogen densities within locally-collected wastewater and greywater (*i.e.*, from decentralized collection systems) were modeled using an epidemiology-based approach (Barker et al., 2013a; Ottoson and Stenström, 2003; Fane et al., 2002; Mok et al., 2014; Barker et al., 2013b). The epidemiology-based approach consisted of three separate phases: 1) estimation of fecal load in the collected water (wet g feces per L water), determined by the ratio of fecal indicator density in the water to that in human feces (both freshly collected); 2) simulation of enteric pathogen infections in the selected population; and 3) estimation of pathogen concentration in the water based on modeled fecal load and the pathogens shed in feces (# ·wet g^−1^) during each simulated infection. Notable model assumptions and their anticipated impact on simulation results are summarized in Supplemental Material. Note that by modeling fecal contamination of water separately from pathogen shedding, we did not assume that fecal indicators are correlated to pathogen occurrence. Refer to Supplemental Material for comparison to the conventional wastewater ratio method (Deere et al., 2006; NRMMC-EPHC-AHMC, 2006; Maimon et al., 2010), which does make this assumption. Also provided in Supplemental Material are a simplified constant pathogen shedding model (Barker et al., 2013a; Ottoson and Stenström, 2003; Mok et al., 2014) and models based on alternative indicators of fecal contamination (Ottoson and Stenström, 2003), as discussed below, for comparison to our simulation.

### Fecal loading

The fecal contamination of onsite/local wastewaters (wet g ·L^−1^) was estimated based on the ratio of previously reported fecal indicator concentrations in the waters (# ·L^−1^) to the indicator content of raw human feces (# ·wet g^−1^). A literature review was conducted to identify studies reporting measurements of fecal indicators in fresh (*i.e.*, not stored) greywater from various household sources (laundry, bathroom sink, and shower/bath water) as well as in domestic wastewater including toilets. Studies from the United States, Australia, Europe, and Israel within the last 25 years were considered in the analysis. To capture variability that may occur among different collection schemes, data from single- or multi-household systems with or without children were included. Since data for other indicators were limited, fecal contamination estimates were based on *Escherichia coli*; where *E. coli* data were unavailable, fecal coliforms were substituted as a conservative surrogate. For comparison, models based on the available data for alternative indicators (coliphages and *Clostridium perfringens*; Ottoson and Stenström (2003)) were also developed; see Supplemental Material. *E. coli* concentration in feces was represented by a PERT distribution (Vose, 2008) following Barker et al. (2013b) (min 7.0, mode 7.4, max 7.9 log_10_ CFU ·L^−1^) (Feachem et al., 1983).

For each water source, a hierarchical model of indicator concentrations was developed. The study results deemed most representative of likely conditions based on methods robustness, sample size, and data reporting were selected to form the base *E. coli* concentration model. This model was constructed by fitting a lognormal distribution to reported summary statistics (median or mean and standard deviation); refer to Supplemental Material for methods used to estimate lognormal parameters (Mood et al., 1974). The lognormal distribution is typically used to represent positive right-skewed data such as microbiological counts (Limpert et al., 2001), and has been previously used to describe indicator concentrations in combined greywater (Ottoson and Stenström, 2003). To include variability amongst studies, the mean parameter of the lognormal distribution was also characterized based on reported mean or median concentration from other studies and standard deviation from the original base model. The resulting range of fitted means was then represented as a PERT distribution about the base model estimate, thereby generating a family of lognormal distributions for each wastewater source. Efforts to perform other types of meta-analysis were limited by the small number of includable studies, which had inconsistent methods between them and results that spanned orders of magnitude. Laundry water was considered to be an arithmetic average of wash and rinse cycles.

Combined greywater from bathroom sinks, showers/baths, and laundry was simulated based on their relative water use in single-family households as reported in the Residential End Uses of Water Study, Version 2 (DeOreo et al., 2016). Consideration of per-household use, rather than per-capita use, accounted for end use that may not scale linearly with number of occupants. Household use of each greywater source (gal ·household^−1^ ·d^−1^) was represented as a lognormal distribution fitted to reported summary statistics (Supplemental Material), as previous investigations have shown this to describe water use characteristics well (Wilkes et al., 2005).

### Infection simulation

Reference enteric pathogens included human-infectious viruses (adenoviruses, *Norovirus*, and *Rotavirus)*, bacteria (*Campylobacter* spp. and *Salmonella enterica)*, and parasitic protozoa (*Cryptosporidium* and *Giardia* spp.), which are anticipated to occur in household wastewaters with the potential to cause human illness (WHO, 2006a). The occurrence of pathogens in greywater was simulated based on population infection incidence rate (infections ·person^−1^ ·year^−1^) and infection duration (days ·infection^−1^) distributions previously reported (Table 2). With the exception of adenoviruses, incidence rates were based on the total number of annual illnesses (2000–2008) estimated by the U.S. Centers for Disease Control and Prevention to be caused by each reference pathogen (Scallan et al., 2011); the model thus neglects seasonality (incidence rates are reported on an annual basis) and asymptomatic infections (illness rates were used as a surrogate for infections). Adenoviruses were not included by Scallan et al., (2011) and infection rate was based on community incidence from Hall et al. (2011). Infection durations and shedding rates were adopted from reviews by Ottoson and Stenström (2003), Gerba (2000), Petterson et al. (2016), Schönning et al. (2007). Based on *Norovirus* GI challenge studies by Atmar et al. (2008), *Norovirus* shedding was modeled as a two-phase distribution with initial high shedding during the first two weeks followed by lower shedding for an extended duration.

In contrast to other studies that have averaged infection incidence over time (Barker et al., 2013a; Ottoson and Stenström, 2003; Mok et al., 2014), which results in an unrealistic fractional number of infections on each day, we developed a probabilistic simulation of daily infections as a modified compound binomial process (Gerber, 1988); comparison to a simplified constant shedding model is provided in Supplemental Material. For each population size considered (5-, 100-, or 1000-person), the number of new pathogen infections on each day of 10,000 possible years was simulated based on the binomial distribution of a daily per-person probability of new infection (annual per-person incidence rate sample/365 days) and population size. The binomial distribution models the discrete number of successes (infections) in a given number of trials (persons in the population) based on the probability of success in each trial (per-person incidence rate) (Vose, 2008). In this approach we assumed that each new infection was independent (no secondary transmission occurs within the selected population) and that there was no person-to-person variability in infection susceptibility (modeled variation in incidence rates reflects uncertainty of the estimated average within 90th percentile credible intervals (Scallan et al., 2011)). Each new infection lasted for a randomly sampled duration (Table 2), and the total number of infected individuals per day was determined by adding together any overlapping infections. Infections persisting at the end of one year continued into the following year. *Norovirus* infections in their first two weeks were differentiated from those in extended phases to facilitate the two-phase concentration model.

### Pathogen concentrations

Daily pathogen concentrations in the various water sources (*P_W_*; # ·L^−1^) were estimated by random sampling of pathogen fecal density (*P_F_*; # ·wet g^−1^; refer to Table 2 for pathogen measurement basis), indicator fecal density (*I_F_; E. coli* CFU ·wet g^−1^), and indicator water concentration (*I_W_; E. coli* CFU ·L^−1^) distributions for each infection *i* occurring on that day (if any) and accounting for dilution effects by wastewater from non-infected individuals:

(1) $$P_{W}=\left(\sum_{i=1}^{N}\frac{P_{F,i}I_{W,i}}{I_{F,i}}\right)\;\left(\frac{1}{\mathrm{Pop}}\right)$$

where *N* is the total number of daily infections from the infection simulation and *Pop* is the population size. *Norovirus* concentrations resulting from the two shedding phases were added together for each day. Infection and pathogen concentration simulations were performed using R 3.2.3 (R Core Team, 2015). Model stability was confirmed by agreement of average daily pathogen infection rates with reported averages and convergence of infection and concentration results between subsequent model runs (overall averages and quantiles by year; 5% tolerance).

Sensitivity analysis for a subset of pathogens (*Campylobacter, Cryptosporidium*, and *Norovirus* spp.) in combined greywater was performed using Spearman’s rank-order correlation through the mc2d package in R (Pouillot and Delignette-Muller, 2010). Parameters included pathogen fecal densities, *E. coli* concentrations in feces and combined greywater, and predicted distributions of total daily infections. Analyses were conducted for each population size (5-, 100-, or 1000-person), both for the overall pathogen concentration results and for the subset of results when pathogens occurred. Since each simulated infection affected multiple days in the model, raw infection incidence rates and shedding durations could not be correlated directly to daily greywater concentrations. An additional analysis examined sensitivity of the combined greywater *E. coli* model to individual source concentrations and household fixture usages.

## Results and discussion

### Quality of locally-collected wastewaters

Greywater quality is inherently variable, both within and between systems, due to specific fecal contamination characteristics of the water source and variation in user behavior (Jefferson et al., 2004; Nolde, 2000). This is evident in the reviewed studies of fecal indicator concentrations (Table 3), wherein reported standard deviations are greater in magnitude than sample means and median values fall orders of magnitude below the mean. Given that concentration is a zero-bounded variable, this indicates right-skewed distributions characterized by many low values and an upper tail of occasional very high values. To capture such variability, the study used lognormal distributions (Table 4) to describe overall distribution shapes and hierarchically nested mean concentration distributions to account for inter-study variability in reported values, rather than relying on a single set of estimates.

Fitted models of *E. coli* in freshly-collected greywater and wastewater, as well as median and 95th percentile simulated concentrations (n = 10,000 simulations), are shown in Table 4. Simulated values are representative of the ranges seen in source studies (Table 3), indicating that the model performed well at characterizing variation in greywater quality. Consistent with Friedler (2004), shower/bath water was the largest source of fecal contamination; Gerba (2000) estimates that an average of 0.14 g of feces are added to water during bathing activity, with potentially up to 10 g added by children. Laundry was generally low in modeled *E. coli* (Table 4) but characterized by high peak concentrations, as may be anticipated to occur during washing of diapers or other highly soiled materials (Nolde, 2000); small low-end concentrations in modeled laundry water result from consideration of non-detects (37–46%) in the O’Toole et al. (2012) study.

Median concentration of *E. coli* in combined greywater (Table 4) was slightly greater than that of individual source streams due to the right-skewed concentration distributions. On average, the contributions of laundry, showers/baths, and sinks to overall greywater volume were 29%, 38%, and 33%, respectively, although bathroom sink usage is overestimated by kitchen sinks and other faucet types being included in the available data (DeOreo et al., 2016). Comparing the different greywater sources, the combined greywater *E. coli* model was most sensitive to modeled shower concentration (Spearman’s *ρ* = 0.73; other sources 0.27–0.30); of the sources considered, shower water had the highest *E. coli* concentrations (Table 4) and thus the greatest influence on variability in combined greywater quality. Water volume contributions from each fixture did not have a meaningful impact on combined greywater quality (|*ρ*| = 0.00–0.04) since relative usages were similar among the sources.

Household wastewater *E. coli* was modeled based on densities measured in influent to domestic onsite wastewater treatment and disposal systems (*i.e.*, septic tanks) (Lowe, 2007; Lowe et al., 2010), and was approximately 2.5 orders of magnitude higher than in modeled combined greywater (Table 4). This is consistent with the World Health Organization’s guidance that greywater be considered a 100–1000 fold wastewater dilution (WHO, 2006b). In addition to greywater sources, total wastewater contains fecal loading from toilets and water from kitchen sinks that can be contaminated during food preparation (WHO, 2006b; Casanova et al., 2001). Concentrations in local wastewater were comparable to municipal wastewater (6.7–8 log_10_ MPN ·100 mL^−1^; Rose et al. (2004)), but exhibited greater variability consistent with lack of wastewater dilution effects in the locally-collected water during peak contamination periods.

Since coliform bacteria have been demonstrated to grow in greywater collection systems (Ottoson and Stenström, 2003; Dixon et al., 2000; Rose et al., 1991), reviewed studies were limited to those reporting fresh (not stored) greywater. Such growth is likely differential from pathogen growth or decay, particularly for non-bacterial pathogens that do not amplify in the environment, such as enteric viruses, and would thus result in poor estimates of pathogen density in the water (O’Toole et al., 2014; WHO, 2006b). Note that *E. coli* was used for estimation of fecal loading to greywater (g feces ·L^−1^ water), rather than as a pathogen surrogate or indicator of pathogen presence; the occurrence and densities of pathogens in such feces was modeled separately. This avoids inaccuracies of the traditional pathogens-to-indicator ratio approach, as discussed in detail by O’Toole et al. (2014), particularly since pathogen detection has been unassociated with levels of indicator bacteria in greywater (O’Toole et al., 2012).

### Epidemiology-based model

Results of the infection simulations are summarized in Table 5. As anticipated, infections rarely occurred in the smallest population studied (5-person); indeed, pathogens with annual per-person incidence rates *<* 0.01 did not appear even during 95th percentile years (5 persons × 0.01 per person per year = 5% annual probability of occurring). The more prevalent pathogens (adenoviruses, *Norovirus*, and *Rotavirus)* appeared within the majority of years as the population size increased to 100-person, and in the 1000-person population all pathogens routinely occurred within a given year. Note that each infection was also simulated to persist for a sampled duration (Table 2) and that in larger populations multiple infections were likely to overlap. Mean per-person annual infection rates for each population size studied were similar and agreed well with input parameters (Table 2). The ability to reproduce reported averages while accounting for the distribution of infections across time highlights the model’s advantage for simulating pathogen occurrence in local collection systems, for which scaling effects are important to the understanding and management of associated health risks (Fane et al., 2002). The infrequent simulated occurrence of pathogens in small populations also justifies their unsuccessful detection in previous studies of greywater quality (Benami et al., 2015; Christova-Boal et al., 1996; Winward et al., 2008), supporting value of the modeling approach.

An important limitation of our epidemiology-based model is the use of illness rates as a surrogate for infection; since only a fraction of infections lead onto disease, the number of days with infections is clearly underestimated. The actual infection rate may be similar to upper 90th percentile credible intervals reported by Scallan et al. (2011), which were approximately double the estimated mean (Table 2). If included, secondary transmission would also impact model results, particularly for pathogens with high secondary spreading potential such as *Norovirus*. Although overall incidence rates would remain the same, occurrences would be clustered as infections are spread among individuals in close proximity. This would likely result in significant overlap of infections, and thus fewer infection days yet higher concentrations on those days. Barker et al. (2013b) modeled pathogen concentrations in wastewater from a small population (approximately 50–100 persons) during outbreak conditions, which included secondary transmission, and found that they were 2–5 orders of magnitude greater than those anticipated in municipal wastewater.

### Pathogen simulation

**Fig. 1:**
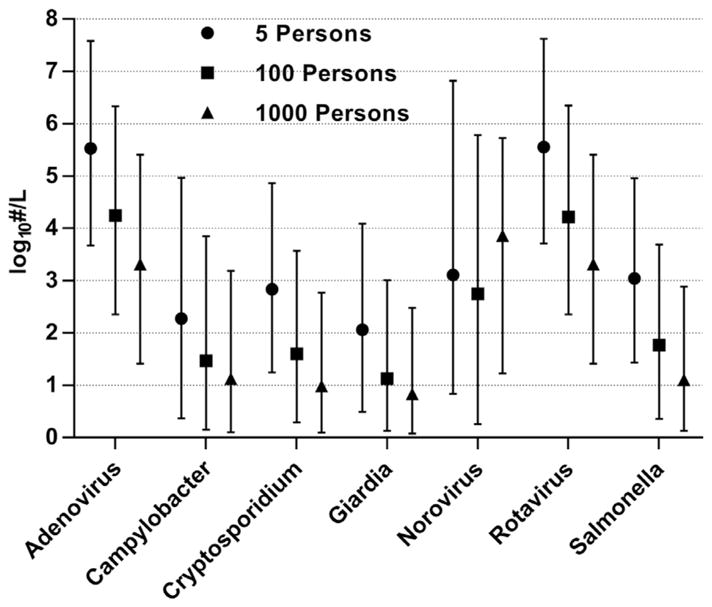
Simulated pathogen concentrations when occuring in combined greywater. Symbols represent median values; whiskers indicate 5th and 95th percentiles. Refer to Table 2 for pathogen measurement basis.

**Fig. 2:**
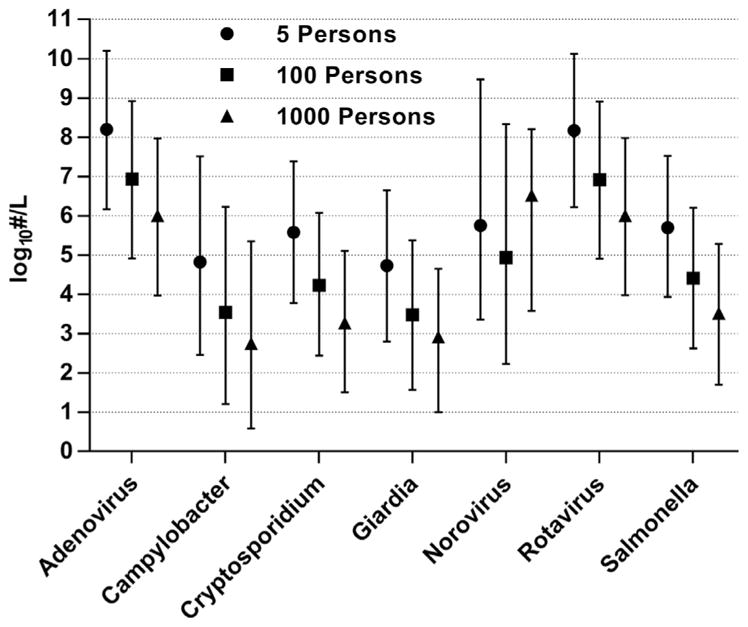
Simulated pathogen concentrations when occuring in local wastewater from all sources including toilets. Symbols represent median values; whiskers indicate 5th and 95th percentiles. Refer to Table 2 for pathogen measurement basis.

The viral reference pathogens, which have both the greatest infection incidence rates and concentrations in feces (Table 2), demonstrated the highest concentrations in modeled greywater (Table 6 and Fig. 1) and wastewater (Table 7 and Fig. 2) (mean concentrations of approximately 5–6 and 7–8 log_10_ ·L^−1^, respectively); refer to Supplemental Material for all water sources. Note, however, that virus particles detected by molecular or microscopic techniques are not necessarily infective; these values should be considered conservative estimates of infectious units. Results do not account for thermal inactivation of pathogens from the use of hot water, nor for chemical disinfection from residual chlorine in supplied water and the use of antimicrobial cleaning products. O’Toole et al. (2012) used qualitative PCR methods to monitor enteric viruses in greywater from single households (bathroom sink n = 36 and washing machine n = 75), with detection rates of 12% and 1% for *Norovirus* (genogroups GI and GII combined) and *Rotavirus*, respectively (Table 1). These rates are somewhat higher than predicted by the 5-person model, but lower than mid-scale (100-person). Modeled concentrations of *Norovirus* in local wastewater (Table 7 and Fig. 2) were higher than estimated for municipal wastewater (mean 3.9 log_10_ copies ·L^−1^) (Pouillot et al., 2015), which may be attributable to greater sensitivity of detection in fresh fecal samples of infected individuals *vs*. dilute municipal wastewater. It should be noted that we used a two-phase shedding model for *Norovirus*, in distinction from others based only on a single peak in shedding (Mok et al., 2014; Barker, 2014), to more accurately represent excretion patterns observed in the original feeding trials by Atmar et al. (2008). This two-phase shedding resulted in mean *Norovirus* concentrations approximately 1–2 orders of magnitude lower than would be predicted using only the peak shedding rate.

Bacterial (*Campylobacter* and *Salmonella* spp.) and parasitic protozoan (*Giardia* and *Cryptosporidium* spp.) pathogens had mean concentrations of approximately 2 log_10_ ·L^−1^ in combined greywater (Table 6; see Supplemental Material for individual sources) and 4 log_10_ ·L^−1^ in local wastewater (Table 7). Bacterial concentrations were based on reported cultivation-based measurements in human feces, which may underestimate infectious populations due to selection bias and presence of viable but nonculturable cells (Oliver, 2005). We identified only two studies reporting enteric pathogen quantities in residential greywater (Table 1). Birks and Hills (2007) measured 0.5–1.5 *Giardia* spp. cysts ·L^−1^ (63% detection, n = 8 samples) in combined greywater from baths, showers, and sinks in 18 university apartments, approximately 1-log order lower than the median mid-scale (100-person) simulation of this study. Given the high detection rate and extended shedding duration of *Giardia* cysts (mean 90 days; Table 2), comparable to the study period of approximately 3 months, it is possible that these results reflect a single infected individual. One sample was positive for *S. enterica* Veltereden (unquantified), but no *Cryptosporidium, Campylobacter, E. coli* O157: H7, or enteroviruses were detected. Kim et al. (2009) reported 5400 *S. enterica* Typhimurium CFU ·100mL^−1^ in greywater from 170 apartments; however, specific water sources, sample size, and detection rates were not stated. Modeled bacterial and protozoan pathogens in local wastewater (Table 7 and Fig. 2) were near the upper range reported in municipal wastewater (*Campylobacter* spp. 3.0–4.6 log_10_ CFU ·L^−1^; *Cryptosporidium* spp. 0.3–4.7 log_10_ oocysts ·L^−1^) (Crockett, 2007; Nasser, 2015; Stampi et al., 1993).

Overall our results demonstrate the impact of system scale on pathogen concentrations: mean concentrations remain the same within water type (Tables 6 and 7, respectively), but the distribution of daily concentrations changes considerably with user population scale (Figs. 1 and 2). With the exception of *Norovirus*, which reached nearly constant occurrence in the 1000-person simulation and no longer displayed scaling effects, median pathogen concentrations in combined greywater (when occurring) decreased by approximately one order of magnitude from 5- to 100-person populations (range 0.8–1.3 log_10_ ·L^−1^) and by 0.3–0.9 log_10_ ·L^−1^ from 100- to 1000-person (Fig. 1); similar trends were observed in local wastewater (Fig. 2). In smaller populations, pathogens rarely occurred; when they did, concentrations were high from lack of wastewater dilution by uninfected individuals. As population size increased there was a greater probability of infection occurring, but dilution effects reduced concentrations. Such effects are evident in occurrence rates and 95th percentiles presented in Tables 6 and 7. The ability to represent this anticipated trend highlights the value of the distributed infection approach over averaging methods, which overestimate occurrence in small populations while underestimating concentrations. For comparison, Barker et al. (2013a) modeled noroviruses in household greywater by averaging monthly incidence rates over each day of the month, estimating median concentrations of −0.3–1.9 log_10_ copies ·L^−1^ (including 25% zeros during the 3 months in which no norovirus infections occurred).

Given the intermittency of pathogen infections, overall model results (*i.e.*, greywater concentrations including both pathogen occurrences and non-occurrences) were most sensitive to the distributions of total daily infections. However, the magnitude of these dependencies varied by pathogen and population size. For less frequently occurring pathogens (*Campylobacter* and *Cryptosporidium* spp.) and for *Norovirus* in the 5- and 100-person simulations, the number of daily infections and greywater concentrations were highly correlated (Spearman’s *ρ* = 0.93–1.00); since the majority of days had zero infections (Table 5), the occurrence of an infection in these datasets had a dominant effect. Note that since such infections rarely overlapped, on days when infections occurred their total number was typically one and thus assigned an equal rank in the analysis. For *Norovirus* in the 1000-person simulation, which reached nearly 100% occurrence (Table 5), the strength of this correlation decreased (*ρ* = 0.23) since the presence of an infection was no longer a distinguishing characteristic. Rather, the relationship was driven by variation in the number of overlapping infections. In the 1000-person *Norovirus* simulation, greywater *E. coli* concentration and pathogen shedding density became more important factors (*ρ* = 0.57 and 0.59, respectively), as was the case for all pathogens and population sizes when considering only days on which pathogens occurred (*ρ* = 0.29–0.76 and 0.15–0.80, respectively). Concentrations of *E. coli* in greywater and of pathogens in feces had comparable effects on variation in greywater pathogen concentration.

### Implications and future needs

The intent of the approaches developed is to support the determination of required pathogen log-reduction values for greywater reuse at various local scales. Since treatment goals are based on annual acceptable risk benchmarks (*e.g.*, 10^−4^ infections ·person^−1^ ·year^−1^), simulation of concentration variation over 10,000 possible years generated a rich dataset for examining the complexities of risk management at these scales. While daily risks may often be low, annual risks will be governed by intermittent high concentrations during infection events. Simulation by year, rather than of independent days to be randomly sampled when generating years of aggregate risk, also allows risk assessors to represent annual risk more realistically by accounting for infections that last multiple days and the overlap of infections within a population. Development of pathogen log-reduction requirements for various combinations of source waters, reference pathogens, and reuse applications based on model outputs are presented in an accompanying article (Schoen et al., 2016). The resulting recommendations should be beneficial to system operators, regulators, and other stakeholders seeking guidance on treatment requirements for decentralized water reuse. In addition to the statistics reported herein, simulation results will be made available for download via the U.S. EPA ScienceHub for further Monte Carlo risk analysis or other modeling effort s.

This research emphasizes the distinction of local wastewater collections from centralized systems in terms of pathogen occurrence and concentration. As such, it highlights the value that would be provided by additional data collected at these scales; however, improved sample collection and analysis methods will be required to increase measurement sensitivities and understanding of infectivity. Results of this work could inform sampling strategies and study design, *e.g.*, power analysis (Cohen, 1992); additional descriptive statistics are provided in Supplemental Material to facilitate such efforts. These initiatives are best directed towards viral pathogens such as *Norovirus* that occur frequently in the population and are shed at high densities, particularly given their important clinical relevance (Ahmed et al., 2014; Hall et al., 2013). For small scales, such as single households, it is unlikely that sampling campaigns will capture sufficient pathogen occurrences to appropriately characterize these waters; such efforts may, therefore, focus on quantifying indicators of fecal contamination to support epidemiology-based modeling as presented here. To that effect, improved characterization of pathogen shedding (including their virulence and fate in water systems) and population infection dynamics (*e.g.*, latency and incubation time, heterogeneity in host interaction, and community transmission) as well as spatial/temporal variability (Haas, 2015), would provide more accurate and, if desired, case-specific wastewater estimates.

Data generated by the current analysis does not include pathogen growth or decay during storage, as only fresh wastewaters were considered, and it does not reflect residual contamination that may persist in closed-loop water reuse systems. Also, not addressed in our work but of potential concern are the water-based (saprozoic) pathogens that may grow post-treatment within plumbing biofilms, such as *Legionella pneumophila, Pseudomonas aeruginosa, Mycobacterium avium* complex, and various fungi (Ashbolt, 2015), that best management practices need to control (*e.g.*, ASHRAE (2015)). Lastly, endotoxins generated from Gramnegative bacteria may also be of concern (Barker et al., 2016), but were not addressed.

### Conclusion

Pathogen concentrations in locally-collected wastewaters are required for the understanding and management of human health risks associated with decentralized water reuse. However, efforts to measure fecal pathogens in onsite greywater have been largely unsuccessful, given low concentrations/non-detects resulting from the sporadic nature of infections in small populations. We propose an epidemiology-based approach to estimate pathogen concentrations that improves upon previously described methods. By characterizing the variability of pathogen occurrence and density in local wastewaters, the developed model provides insight into scaling effects that occur in small greywater and wastewater collection systems, such as single households, apartment buildings, or subdivisions, and further emphasizes their distinction from centralized municipal wastewater. In addition to differences in anticipated quality between greywater and local wastewater, simulation results demonstrate that pathogen occurrences in the various wastewaters are intermittent at these collection scales, increasing in frequency with user population size. However, when pathogens do occur in small collection systems, concentrations are high from lack of wastewater dilution effects. Quantitative representation of the interplay between these trends provides a valuable tool for analyzing the risks associated with reuse of onsite-collected waters.

## Supplementary Material

data

## Figures and Tables

**Table 1:** Reported enteric reference pathogen measurements in greywater (GW).

Pathogen genus	Sample type	Sample size	Occurrence	Concentration range	Reference
*Campylobacter*	Laundry	Not stated	Not detected	Not detected	Christova-Boal et al. (1996)
*Cryptosporidium*	Laundry	Not stated	Not detected	Not detected	Christova-Boal et al. (1996)
*Giardia*	Laundry	Not stated	Not detected	Not detected	Christova-Boal et al. (1996)
*Norovirus*	Laundry	75	13%	Qualitative only	O’Toole et al. (2012)
*Rotavirus*	Laundry	75	1%	Qualitative only	O’Toole et al. (2012)
*Salmonella*	Laundry	Not stated	Not detected	Not detected	Christova-Boal et al. (1996)
*Campylobacter*	Shower/bath	Not stated	Not detected	Not detected	Christova-Boal et al. (1996)
*Cryptosporidium*	Shower/bath	Not stated	Not detected	Not detected	Christova-Boal et al. (1996)
*Giardia*	Shower/bath	Not stated	Not detected	Not detected	Christova-Boal et al. (1996)
*Norovirus*	Shower/bath	36	8%	Qualitative only	O’Toole et al. (2012)
*Rotavirus*	Shower/bath	36	Not detected	Not detected	O’Toole et al. (2012)
*Salmonella*	Shower/bath	Not stated	Not detected	Not detected	Christova-Boal et al. (1996)
*Campylobacter*	Bathroom sink	3	Not detected	Not detected	Birks et al. (2004)
*Cryptosporidium*	Bathroom sink	3	67%	0.4–1.2 oocysts ·L^−1^	Birks et al. (2004)
*Giardia*	Bathroom sink	3	67%	0.6–1.2 cysts ·L^−1^	Birks et al. (2004)
*Salmonella*	Bathroom sink	3	Not detected	Not detected	Birks et al. (2004)
*Campylobacter*	Combined GW	9	Not detected	Not detected	Winward et al. (2008)
*Campylobacter*	Combined GW	8	Not detected	Not detected	Birks and Hills (2007)
*Cryptosporidium*	Combined GW	8	Not detected	Not detected	Birks and Hills (2007)
*Giardia*	Combined GW	8	63%	0.5–1.5 cysts ·L^−1^	Birks and Hills (2007)
*Salmonella*	Combined GW	13	Not detected	Not detected	Winward et al. (2008)
*Salmonella*	Combined GW	9	Not detected	Not detected	Benami et al. (2015)
*Salmonella*	Combined GW	8	13%	Not stated	Birks and Hills (2007)
*Salmonella*	Combined GW	Not stated	Not stated	5400 CFU ·100mL^−1^	Kim et al. (2009)

**Table 2:** Reported distributions of pathogen densities in feces, shedding durations, and population infection incidence rates.

Pathogen genus	Parameter	Units	Distribution	Distribution values			Reference
Adenoviruses	Density	log_10_ particles ·wet g^−1^	Triangle (min, mode, max)	8	10	12	Petterson et al. (2016)
Adenoviruses	Duration	days	Triangle (min, mode, max)	3	7	12	Petterson et al. (2016)
Adenoviruses	Incidence	10^4^p^−1^·y^−1^	PERT (min, mode, max)	41	97	210	Hall et al. (2011)
*Campylobacter*	Density	log_10_ CFU ·wet g^−1^	Triangle (min, mode, max)	4	6	10	Petterson et al. (2016)
*Campylobacter*	Duration	days	Triangle (min, mode, max)	15	34	42	Petterson et al. (2016)
*Campylobacter*	Extreme duration^a^	proportion of cases	Triangle (min, mode, max)	0.005	0.0075	0.01	Petterson et al. (2016)
		days	Point estimate	60			
*Campylobacter*	Incidence	10^4^p^−1^·y^−1^	PERT (min, mode, max)	14	35	68	Scallan et al. (2011)
*Cryptosporidium*	Density	log_10_ oocysts·wet g^−1^	Triangle (min, mode, max)	6	7	9	Petterson et al. (2016)
*Cryptosporidium*	Duration	days	Triangle (min, mode, max)	5	10	30	Petterson et al. (2016)
*Cryptosporidium*	Extreme duration	proportion of cases	Point estimate	0.02			Petterson et al. (2016)
		days	Point estimate	60			
*Cryptosporidium*	Incidence	10^4^p^−1^·y^−1^	PERT (min, mode, max)	5	23	65	Scallan et al. (2011)
*Giardia*	Density	ln cysts ·wet g^−1^	Normal (mean, sd)	15	1.7		Schönning et al. (2007)
*Giardia*	Duration	ln days	Normal (mean, sd)	4.5	0.7		Schönning et al. (2007)
*Giardia*	Incidence	10^4^p^−1^·y^−1^	PERT (min, mode, max)	27	38	50	Scallan et al. (2011)
*Norovirus*	Density	log_10_ gc·wet g^−1^	PERT (min, mode, max)	7.5	9.75	12	Atmar et al. (2008)
*Norovirus*	Extended density^b^	log_10_ gc·wet g^−1^	PERT (min, mode, max)	3.5	6.5	7.6	Atmar et al. (2008)
*Norovirus*	Duration	days	PERT (min, mode, max)	13	28	56	Atmar et al. (2008)
*Norovirus*	Incidence	10^4^p^−1^·y^−1^	PERT (min, mode, max)	428	696	1025	Scallan et al. (2011)
*Rotavirus*	Density	log_10_ particles ·wet g^−1^	Triangle (min, mode, max)	8	10	12	Petterson et al. (2016)
*Rotavirus*	Duration	days	Triangle (min, mode, max)	3	7	12	Petterson et al. (2016)
*Rotavirus*	Incidence	10^4^p^−1^·y^−1^	PERT (min, mode, max)	79	103	128	Scallan et al. (2011)
*Salmonella*	Density	log_10_ CFU·wet g^−1^	Triangle (min, mode, max)	6	7.5	9	Petterson et al. (2016)
*Salmonella*	Duration	days	Triangle (min, mode, max)	10	15	50	Petterson et al. (2016)
*Salmonella*	Incidence	10^4^p^−1^·y^−1^	PERT (min, mode, max)	23	37	60	Scallan et al. (2011)

a Based on evidence of extended shedding in a fraction of the population

b Atmar et al. (2008) report initial high-shedding during the first two weeks followed by extended shedding at a lower density

**Table 3:** Studies included in models of *E. coli* in various household water sources; units are log_10_ CFU or log_10_ MPN per 100 mL. Studies used for the base model are identified in bold.

Indicator	Source	n	Mean	SD	Min	Median	Max	Reference
FC	Laundry				2.04		3.04	Christova-Boal et al. (1996)
FC	Laundry	35	6.60					Friedler (2004)
***E. coli***	**Laundry rinse**	**74**	**3.53**	**4.45**	**n/a**^a^	**0.00**	**5.38**	O’Toole et al. (2012)
***E. coli***	**Laundry wash**	**75**	**5.04**	**5.98**	**n/a**	**0.30**	**6.91**	O’Toole et al. (2012)
FC	Laundry rinse	57	1.40					Rose et al. (1991)
FC	Laundry wash	57	2.10					Rose et al. (1991)
FC	Shower/bath				2.23		3.52	Christova-Boal et al. (1996)
FC	Shower/*bath*^b^	10	6.60	6.84				Friedler (2004)
FC	*Shower*/bath	19	6.60	6.93				Friedler (2004)
*E. coli*	Shower/*bath*	34	1.92	2.08				Jefferson et al. (2004)
*E. coli*	*Shower*/bath	34	3.17	3.69				Jefferson et al. (2004)
FC	Shower/bath				1.00		3.00	Nolde (2000)
***E. coli***	**Shower/bath**	**36**	**3.23**	**3.65**	**n/a**	**2.11**	**4.32**	O’Toole et al. (2012)
FC	Shower/bath	57	3.78					Rose et al. (1991)
FC	Shower/bath	8	4.65	4.78				Santos et al. (2014)
FC	Bathroom sink	33	3.54	3.87				Friedler (2004)
*E. coli*	Bathroom sink	34	1.00	3.94				Jefferson et al. (2004)
**FC**	**Bathroom sink**	**8**	**2.52**	**2.72**				Santos et al. (2014)
FC	Local WW	5^c^			4.48	5.69	6.87	Lowe (2007)
***E. coli***	**Local WW**	**57**	**6.52**	**7.04**	**4.00**	**5.48**	**7.91**	Lowe et al. (2010)

Abbreviations: FC, fecal coliforms; SD, standard deviation; WW, wastewater

a Not applicable; some samples below detection limits

b For studies reporting both shower and bath, italics indicate which source

c Literature review; number of studies considered

**Table 4:** Modeled *E. coli* concentration in wastewater from various household sources; percentiles based on 10,000 simulations. Combined greywater (GW) was modeled by relative household water use for each fixture. Local wastewater (WW) represents mixed wastewater including toilets.

Water source	Lognormal parameters (lnCFU ·100mL^−1^)				Modeled concentration (log_10_ CFU ·L^−1^)		
	min μ	mode μ	max μ	*σ*	5%	50%	95%
Laundry wash	−1.70	0.69	10.08	3.61	–	–	–
Laundry rinse	−1.90	0.00	10.08	3.20	–	–	–
Laundry overall	–	–	–	–	−1.1	1.8	4.8
Shower/bath	2.65	4.87	13.43	1.88	1.8	3.5	5.6
Bathroom sink	1.68	5.18	7.54	1.12	2.0	3.2	4.3
Combined GW	–	–	–	–	2.4	3.6	5.3
Local WW	12.10	12.10	13.10	2.03	4.9	6.3	7.8

**Table 5:** Mean annual infection rates and percentiles of annual days with infections occuring (n = 10,000 years) for each population size.

	5-person				100-person				1000-person			
	Mean rate (10^4^p^−1^·y^−1^)	Infection days/year			Mean rate (10^4^p^−1^·y^−1^)	Infection days/year			Mean rate (10^4^p^−1^·y^−1^)	Infection days/year		
		5%	50%	95%		5%	50%	95%		5%	50%	95%
Adenoviruses	107	0	0	4	107	0	7	24	106	40	73	111
*Campylobacter*	33	0	0	0	36	0	0	46	37	29	98	178
*Cryptosporidium*	28	0	0	0	26	0	0	23	27	0	38	91
*Giardia*	37	0	0	0	38	0	0	193	38	87	268	365
*Norovirus*	686	0	0	45	711	79	164	247	706	358	365	365
*Rotavirus*	107	0	0	5	104	0	7	24	103	38	71	108
*Salmonella*	36	0	0	0	38	0	0	43	38	20	83	158

**Table 6:** Simulated pathogen concentrations in combined greywater: rate of occurrence, net mean including non-occurrences, and 95th percentile when occurring. Concentrations are expressed as log_10_ per L; refer to Table 2 for pathogen measurement basis.

	5-person			100-person			1000-person		
	Occurrence	Mean	95th%	Occurrence	Mean	95th%	Occurrence	Mean	95th%
Adenoviruses	0.1%	4.57	7.58	2.3%	4.88	6.34	20.3%	4.85	5.41
*Campylobacter*	0.1%	2.25	4.97	3.1%	2.55	3.85	27.3%	2.85	3.19
*Cryptosporidium*	0.1%	1.83	4.87	1.2%	1.85	3.57	11.3%	1.87	2.77
*Giardia*	0.6%	2.05	4.09	11.3%	2.08	3.01	69.8%	2.02	2.48
*Norovirus*	2.8%	5.82	6.82	44.8%	5.63	5.78	99.7%	5.93	5.73
*Rotavirus*	0.1%	4.59	7.62	2.2%	4.66	6.35	19.8%	4.82	5.41
*Salmonella*	0.1%	1.93	4.96	2.6%	2.34	3.69	23.2%	2.16	2.89

**Table 7:** Simulated pathogen concentrations in local wastewater from all sources including toilets: rate of occurrence, net mean including non-occurrences, and 95th percentile when occurring. Concentrations are expressed as log_10_ per L; refer to Table 2 for pathogen measurement basis.

	5-person			100-person			1000-person		
	Occurrence	Mean	95th%	Occurrence	Mean	95th%	Occurrence	Mean	95th%
Adenoviruses	0.1%	6.93	10.20	2.3%	6.84	8.92	20.3%	6.86	7.97
*Campylobacter*	0.1%	4.43	7.51	3.1%	4.49	6.23	27.3%	4.53	5.35
*Cryptosporidium*	0.1%	3.59	7.38	1.2%	3.72	6.07	11.3%	3.71	5.11
*Giardia*	0.6%	4.00	6.65	11.3%	4.07	5.38	69.8%	4.06	4.65
*Norovirus*	2.8%	7.66	9.47	44.8%	7.70	8.34	99.7%	7.70	8.21
*Rotavirus*	0.1%	6.93	10.12	2.2%	6.83	8.91	19.8%	6.88	7.98
*Salmonella*	0.1%	4.12	7.52	2.6%	4.16	6.20	23.2%	4.16	5.28
