# Supplementary material for: Simulation of enteric pathogen concentrations in locally-collected greywater and wastewater for microbial risk assessments
Source: Microb Risk Anal. Author manuscript; Available in PMC 2018 Aug 22. (PMC6104838; doi:10.1016/j.mran.2016.11.001)
Supplement: data [file NIHMS983385-supplement-data.docx]

**Supplemental Material: Simulation of Enteric Pathogen Concentrations in Locally-Collected Greywater and Wastewater for Microbial Risk Assessments**

*Michael Jahne^1^, Mary Schoen^2^, Jay Garland^1^, Nicholas Ashbolt^3^*

^1^ U.S. Environmental Protection Agency, 26 W. Martin Luther King Dr., Cincinnati OH 45268

^2^ Soller Environmental, 3022 King St., Berkeley, CA 94703

^3^ University of Alberta, Rm 3-57D South Academic Building, Edmonton, AB T6G 2G7

**Contents** (18 pages)

**Page S2**: Lognormal Parameter Estimation

**Page S3**: Alternative Indicator Models

**Page S6**: Wastewater Ratio Approach

**Page S8**: Constant Shedding Model

**Page S10**: Notable Model Assumptions

**Page S11**: Simulated Pathogen Concentrations in All Water Sources

**Page S16**: References

**Tables**

**Page S5**: Table S1. Simulated pathogen concentrations in greywater based on coliphage.

**Page S5**: Table S2. Simulated pathogen concentrations in greywater based on *C. perfringens*.

**Page S7**: Table S3. Reported indicator and pathogen densities in municipal wastewater

**Page S7**: Table S4. Simulated pathogen concentrations based on the wastewater ratio approach.

**Page S9**: Table S5. Simulated pathogen concentrations based on the constant shedding model.

**Page S10**: Table S6. Notable model assumptions and anticipated impact on simulation results.

**Page S11**: Table S7. Simulated pathogen concentrations in bathroom sink water.

**Page S12**: Table S8. Simulated pathogen concentrations in bathroom shower/bath water.

**Page S13**: Table S9. Simulated pathogen concentrations in bathroom laundry water.

**Page S14**: Table S10. Simulated pathogen concentrations in combined greywater.

**Page S15**: Table S11. Simulated pathogen concentrations in local wastewater.

**Lognormal Parameter Estimation**

For base model studies reporting sample median and standard deviation (SD) values (*i.e.*, Lowe et al. (2010), Ottoson and Stenström (2003)), lognormal σ was estimated by:

$\sigma=\sqrt{\ln\left( \frac{SD}{Median} \right)}$ [1]

For these studies, and for additional studies reporting sample median (*i.e.*, Lowe (2007)), lognormal µ was estimated by:

$\mu=\ln\left( Median \right)$ [2]

For base model studies reporting sample mean and SD values (*i.e.*, Santos et al. (2014)), lognormal σ was estimated by:

$\sigma=\sqrt{\ln\left( 1+\frac{{SD}^{2}}{{Mean}^{2}} \right)}$ [3]

For these studies, and for additional studies reporting sample mean (*i.e.*, Friedler (2004), Jefferson et al. (2004), Rose et al. (1991)), lognormal µ was estimated by:

$\mu=\ln\left( Mean \right)-\frac{\sigma^{2}}{2}$ [4]

where σ is from the respective base model.

For studies reporting sample range (*i.e.*, Christova-Boal et al. (1996), Nolde (2000)), lognormal µ was estimated by:

$\mu=\frac{\ln\left( Min \right)+\ln\left( Max \right)}{2}$ [5]

For studies reporting sample mean and 95% confidence interval (CI) (*i.e.*, Deoreo et al. (2016)), sample SD was calculated prior to use of formulas [3] and [4] by:

$SD=95\%CI\times\frac{\sqrt{n}}{1.96}$ [6]

where *n* is the sample size.

**Alternative Indicator Models**

For comparison to the *E. coli*-based fecal contamination model, pathogens were also simulated based on concentrations of alternative fecal indicators coliphage and *Clostridium perfringens* in greywater; however, data were limited. The best available estimates were provided by Ottoson and Stenström (2003), who reported log_10_normal distributions of somatic coliphage (mean 3.3 log_10_PFU·100mL^-1^; SD 0.61) and *C. perfringens* (mean 3.3 log_10_CFU·100mL^-1^; SD 0.61) in combined stored greywater from 85 row houses (212 individuals). Associated pathogen concentrations were estimated using methods described in the main text (see "Pathogen Concentrations" section of Methods) based on fecal density PERT distributions of minimum, mode, and maximum 5.4, 5.7, 6.3 log_10_CFU·wet g^-1^ *C. perfringens* (Feachem et al. 1983) and 1, 2.7, 6.7 log_10_PFU·wet g^-1^ somatic coliphage (Havelaar et al. 1990).

Pathogen results (Table S1 and Table S2) were considerably greater than those based on *E. coli* (Table S10), since concentrations of these indicators in greywater were comparable to that observed in local wastewater (3-5 and 3-4 log_10_·100mL^-1^ *C. perfringens* and coliphage, respectively (Lowe 2007)). This may be attributable to overestimation of the indicators in stored greywater of the Ottoson and Stenström (2003) study; other studies of household greywater have failed to detect coliphage (Casanova et al. 2001, Gilboa and Friedler 2008), and the authors note that coliphage may have propagated in their system due to (likely) growth of its coliform host. Although Winward et al. (2008) report similar levels of *C. perfringens*, their study also experienced coliform growth; regrowth of *C. perfringens* in wastewater has likewise been demonstrated (Alonso et al. 2004), and the long persistence of its spores in environmental matrices has limited its widespread use as a fecal indicator (Ashbolt et al. 2001). Gilboa and Friedler (2008) measured considerably lower *C. perfringens* in a combined greywater equalization basin (mean 0.7 log_10_CFU·100mL^-1^ with 86% detection), along with 1-2 log orders lower *E. coli*. Given that levels of alternative indicators in modeled greywater (first rows of Table S1 and Table S2) were similar to *E. coli* (combined greywater in main text Table 4) despite their lower levels in feces, fecal contamination is overestimated by them. Indeed, differences in simulated pathogen concentrations between the indicators directly paralleled differences in their fecal densities (modes of 7.4, 5.7, and 2.7 log_10_·g^-1^ *E. coli*, *C. perfringens*, and somatic coliphage, respectively).

**Table S1.** Simulated indicator and pathogen concentrations in combined greywater based on coliphage: rate of occurrence, percentiles of concentration when occurring, and net mean including non-occurrences. Concentrations are expressed as log_10_ per L.

|  | **5-persons** | | | | | **100-persons** | | | | | **1,000-persons** | | | | |
| --- | --- | --- | --- | --- | --- | --- | --- | --- | --- | --- | --- | --- | --- | --- | --- |
|  | **Occurrence** | **5%** | **50%** | **95%** | **Net Mean** | **Occurrence** | **5%** | **50%** | **95%** | **Net Mean** | **Occurrence** | **5%** | **50%** | **95%** | **Net Mean** |
| *Coliphage* | 100% | 3.25 | 4.30 | 5.32 | 4.75 | 100% | 3.25 | 4.30 | 5.32 | 4.75 | 100% | 3.25 | 4.30 | 5.32 | 4.75 |
| Adenoviruses | 0.1% | 8.02 | 10.56 | 12.83 | 9.62 | 2.3% | 6.74 | 9.26 | 11.57 | 9.56 | 20.3% | 5.82 | 8.37 | 10.63 | 9.52 |
| *Campylobacter* | 0.1% | 4.32 | 7.13 | 10.12 | 7.39 | 3.1% | 3.05 | 5.88 | 8.80 | 7.18 | 27.3% | 2.18 | 5.05 | 7.92 | 7.20 |
| *Cryptosporidium* | 0.1% | 5.61 | 7.85 | 10.03 | 6.26 | 1.2% | 4.24 | 6.58 | 8.73 | 6.36 | 11.3% | 3.27 | 5.65 | 7.77 | 6.36 |
| *Giardia* | 0.6% | 4.62 | 7.12 | 9.32 | 6.62 | 11.3% | 3.37 | 5.84 | 8.04 | 6.72 | 69.8% | 2.81 | 5.35 | 7.32 | 6.72 |
| *Norovirus* | 2.8% | 9.11 | 11.48 | 13.54 | 11.49 | 44.8% | 8.04 | 10.45 | 12.39 | 11.50 | 99.7% | 9.35 | 10.88 | 12.08 | 11.49 |
| *Rotavirus* | 0.1% | 8.00 | 10.53 | 12.93 | 9.54 | 2.2% | 6.75 | 9.27 | 11.56 | 9.53 | 19.8% | 5.82 | 8.37 | 10.63 | 9.51 |
| *Salmonella* | 0.1% | 5.72 | 8.07 | 10.20 | 6.79 | 2.6% | 4.41 | 6.78 | 8.87 | 6.83 | 23.2% | 3.49 | 5.89 | 7.95 | 6.82 |

**Table S2.** Simulated indicator and pathogen concentrations in combined greywater based on *C. perfringens*: rate of occurrence, percentiles of concentration when occurring, and net mean including non-occurrences. Concentrations are expressed as log_10_ per L.

|  | **5-persons** | | | | | **100-persons** | | | | | **1,000-persons** | | | | |
| --- | --- | --- | --- | --- | --- | --- | --- | --- | --- | --- | --- | --- | --- | --- | --- |
|  | **Occurrence** | **5%** | **50%** | **95%** | **Net Mean** | **Occurrence** | **5%** | **50%** | **95%** | **Net Mean** | **Occurrence** | **5%** | **50%** | **95%** | **Net Mean** |
| *C. perfringens* | 100% | 3.32 | 4.30 | 5.29 | 4.75 | 100% | 3.32 | 4.30 | 5.29 | 4.75 | 100% | 3.32 | 4.30 | 5.29 | 4.75 |
| Adenoviruses | 0.1% | 6.15 | 7.82 | 9.52 | 6.03 | 2.3% | 4.86 | 6.56 | 8.26 | 6.05 | 20.3% | 3.90 | 5.64 | 7.31 | 6.03 |
| *Campylobacter* | 0.1% | 2.32 | 4.39 | 6.93 | 3.67 | 3.1% | 1.11 | 3.15 | 5.66 | 3.68 | 27.3% | 0.46 | 2.36 | 4.77 | 3.70 |
| *Cryptosporidium* | 0.1% | 3.75 | 5.16 | 6.62 | 2.85 | 1.2% | 2.47 | 3.86 | 5.38 | 2.86 | 11.3% | 1.48 | 2.90 | 4.41 | 2.87 |
| *Giardia* | 0.6% | 2.78 | 4.38 | 5.98 | 3.17 | 11.3% | 1.50 | 3.11 | 4.70 | 3.23 | 69.8% | 0.87 | 2.50 | 3.94 | 3.23 |
| *Norovirus* | 2.8% | 7.34 | 8.77 | 10.13 | 7.98 | 44.8% | 6.17 | 7.66 | 8.95 | 8.00 | 99.7% | 6.69 | 7.72 | 8.52 | 8.00 |
| *Rotavirus* | 0.1% | 6.07 | 7.86 | 9.63 | 6.09 | 2.2% | 4.86 | 6.56 | 8.26 | 6.02 | 19.8% | 3.90 | 5.64 | 7.31 | 6.02 |
| *Salmonella* | 0.1% | 3.90 | 5.36 | 6.80 | 3.34 | 2.6% | 2.60 | 4.06 | 5.51 | 3.33 | 23.2% | 1.66 | 3.14 | 4.56 | 3.33 |

**Wastewater Ratio Approach**

For comparison, a subset of pathogen concentrations in combined greywater (*P_GW_*, log_10·_L^-1^) were also simulated using the wastewater ratio approach (Deere et al. 2006, NRMMC-EPHC-AHMC 2006, Maimon et al. 2010). Pathogen densities in municipal wastewater (*P_WW_*, log_10_·L^-1^) were weighted by the relative concentrations of *E. coli* in combined greywater (*I_GW_*, MPN·L^-1^; as determined in main text) and municipal wastewater (*I_WW_*, MPN·L^-1^):

$P_{GW}=P_{WW}\left( \frac{I_{GW}}{I_{WW}} \right)$ [7]

Input municipal wastewater distributions (Table S3) were randomly sampled during each of 10,000×365 daily simulations using R 3.2.3 (R Core Team 2015). This method does not account for variation in population size.

Mean concentrations of *Campylobacter* spp. and *Cryptosporidium* spp. determined by the wastewater ratio method (Table S4) were comparable to those from the epidemiology-based simulation (Table S10; all scales). Median and 95^th^ percentiles in the 1,000-person simulation were greater by 0.25-0.82 log orders, and more so in the 5- and 100-person models. Given that these organisms had only 27.3% and 11.3% infection occurrence (Table S10), respectively, in the 1,000-person model, this suggests additional scaling effects beyond 1,000-person systems and highlights value of the epidemiology-based approach for such characterization. Despite high norovirus prevalence rates in the 1,000-person simulation (nearly 100%), which would suggest similarity to municipal wastewater, concentration was notably greater in the epidemiology-based model (2-3 orders of magnitude). This discrepancy may be associated with greater sensitivity of detection in fecal samples of infected individuals vs. dilute municipal wastewater, decay prior to reaching centralized wastewater treatment plants, or inclusion of concentrations below the detection limit for non-detects in the Pouillot et al. (2015) model on which wastewater concentrations were based.

**Table S3.** Reported indicator and pathogen densities in municipal wastewater (Soller et al. 2016).

|  | **Distribution** | **Units** | **Values** | | **Reference** |
| --- | --- | --- | --- | --- | --- |
| *E. coli* | Uniform (min, max) | log_10_CFU·L^-1^ | 6.7 | 8.0 | Rose et al. (2004) |
| *Campylobacter* | Uniform (min, max) | log_10_CFU·L^-1^ | 2.95 | 4.6 | Stampi et al. (1993) |
| *Cryptosporidium* | Uniform (min, max) | log_10_oocysts·L^-1^ | 0.3 | 4.7 | Crockett (2007); Nasser (2015) |
| *Norovirus* | Normal (mean, sd) | log_10_gc·L^-1^ | 3.9 | 1.4 | Pouillot et al. (2015) |

**Table S4.** Simulated pathogen concentrations in combined greywater based on the wastewater ratio approach: percentiles of concentration and net mean. Concentrations are expressed as log_10_ per L; refer to Table S3 for measurement basis.

|  | **Occurrence** | **5%** | **50%** | **95%** | **Net Mean** |
| --- | --- | --- | --- | --- | --- |
| *Campylobacter* | 100% | 0.07 | 0.74 | 2.37 | 2.17 |
| *Cryptosporidium* | 100% | 0.06 | 0.72 | 2.36 | 1.74 |
| *Norovirus* | 100% | 0.12 | 1.22 | 3.56 | 4.00 |

**Constant Shedding Model**

For consistency with other studies using the constant shedding approach (Ottoson and Stenström 2003, Barker et al. 2013, Mok et al. 2014, Fane et al. 2002), pathogen concentrations were also modeled using the simplified constant shedding method. Pathogen concentrations in combined greywater (*P_W_*; #·L^-1^) were estimated by random sampling of pathogen fecal density (*P_F_*; #·wet g^-1^), indicator fecal density (*I_F_*; *E. coli* CFU·wet g^-1^), indicator water concentration (*I_W_*; *E. coli* MPN·L^-1^), daily average per-person infection rate (*IR*; day^-1^), and infection duration (*D*, days) distributions (Table 2 and Table 4 of main text) for each day of 10,000 years:

$P_{W}=\left( \frac{P_{F,i}I_{W,i}}{I_{F,i}} \right)\left( IR \right)\left( D \right)$ [8]

Note that since infection incidence rates are averaged over the same population in which dilution occurs, this model in insensitive to system scale. The fractional shedding model for norovirus was not conducive and was replaced with that by Barker (2014) based on the same Atmar et al. (2008) data (PERT minimum 9.2, mode 11, max 12.2 log_10_gc·wet g^-1^). Monte Carlo simulation was performed using R 3.2.3 (R Core Team 2015).

As expected, mean concentrations determined by this approach (Table S5) agreed well with those from the epidemiology-based approach (Table S10). However, mean concentrations were strongly influenced by rare extreme highs (means comparable to, or greater than, 95^th^ percentile concentrations), with percentiles indicating lower concentrations than when occurring in the 1,000-person simulation and orders of magnitude lower than in the 5-and 100-person models. Given differences in occurrence rates (100% vs. less), these results are consistent with additional scaling effects beyond 1,000-persons. This demonstrates value of the distributed infection approach for characterizing small collection systems; as discussed in the main text, the simplified model overestimates occurrence while underestimating concentrations when occurring.

**Table S5**. Simulated pathogen concentrations in combined greywater based on the constant shedding model: percentiles of concentration and net mean. Concentrations are expressed as log_10_ per L; refer to main text for measurement basis.

|  | **Occurrence** | **5%** | **50%** | **95%** | **Net Mean** |
| --- | --- | --- | --- | --- | --- |
| Adenoviruses | 100% | 0.73 | 2.54 | 4.65 | 4.92 |
| *Campylobacter* | 100% | 0.08 | 0.92 | 2.84 | 2.42 |
| *Cryptosporidium* | 100% | 0.05 | 0.65 | 2.22 | 1.82 |
| *Giardia* | 100% | 0.06 | 0.73 | 2.39 | 2.18 |
| *Norovirus* | 100% | 3.26 | 4.85 | 6.76 | 6.87 |
| *Rotavirus* | 100% | 0.74 | 2.54 | 4.65 | 4.87 |
| *Salmonella* | 100% | 0.07 | 0.78 | 2.44 | 2.29 |

**Table S6.** Notable model assumptions and anticipated impact on simulation results.

| **Assumption** | **Justification** | **Potential Impact** | **Relative Importance** |
| --- | --- | --- | --- |
| Fecal coliforms are a conservative surrogate for *E. coli* | Availability of data | Overestimation of concentrations | Low |
| Infections spread evenly throughout year | Availability of data; model simplification | Underestimation of peak concentrations; overestimation of occurrence frequency | Medium |
| Illness rates representative of infections | Availability of data | Underestimation of concentrations and occurrence frequency | Medium |
| Independent infections (no secondary transmission) | Availability of data; model simplification | Underestimation of peak concentrations; overestimation of occurrence frequency | Medium |
| No variability in infection susceptibility of individuals | Availability of data | Over- or underestimation of concentrations and/or occurrence frequency | Low |
| Lognormal input distributions | Previous literature; model simplification | Over- or underestimation of concentrations | Medium |

**Simulated Pathogen Concentrations in All Water Sources**

**Table S7.** Simulated pathogen concentrations in bathroom sink water: rate of occurrence, percentiles of concentration when occurring, mean and standard deviation (SD) when occurring. Concentrations are expressed as log_10_ per L; refer to main text for measurement basis.

|  | **5-persons** | | | | | | **100-persons** | | | | | | **1,000-persons** | | | | | |
| --- | --- | --- | --- | --- | --- | --- | --- | --- | --- | --- | --- | --- | --- | --- | --- | --- | --- | --- |
|  | **Occurrence** | **5%** | **50%** | **95%** | **Mean** | **SD** | **Occurrence** | **5%** | **50%** | **95%** | **Mean** | **SD** | **Occurrence** | **5%** | **50%** | **95%** | **Mean** | **SD** |
| Adenoviruses | 0.1% | 3.30 | 5.05 | 6.86 | 5.05 | 1.07 | 2.3% | 1.98 | 3.77 | 5.52 | 3.76 | 1.07 | 20.3% | 1.06 | 2.85 | 4.58 | 2.84 | 1.06 |
| *Campylobacter* | 0.1% | 0.26 | 1.82 | 4.23 | 2.00 | 1.24 | 3.1% | 0.11 | 1.19 | 3.23 | 1.37 | 0.98 | 27.3% | 0.08 | 0.90 | 2.54 | 1.05 | 0.78 |
| *Cryptosporidium* | 0.1% | 0.91 | 2.37 | 3.97 | 2.40 | 0.93 | 1.2% | 0.17 | 1.22 | 2.72 | 1.30 | 0.78 | 11.3% | 0.07 | 0.70 | 1.94 | 0.81 | 0.59 |
| *Giardia* | 0.6% | 0.32 | 1.66 | 3.27 | 1.71 | 0.90 | 11.3% | 0.08 | 0.82 | 2.18 | 0.93 | 0.66 | 69.8% | 0.05 | 0.53 | 1.59 | 0.64 | 0.49 |
| *Norovirus* | 2.8% | 0.56 | 2.52 | 6.17 | 3.01 | 1.84 | 44.8% | 0.19 | 2.51 | 5.11 | 2.47 | 1.65 | 99.7% | 0.87 | 3.34 | 4.77 | 3.19 | 1.12 |
| *Rotavirus* | 0.1% | 3.28 | 5.04 | 6.83 | 5.03 | 1.08 | 2.2% | 1.98 | 3.75 | 5.51 | 3.75 | 1.08 | 19.8% | 1.06 | 2.85 | 4.58 | 2.84 | 1.06 |
| *Salmonella* | 0.1% | 1.01 | 2.56 | 4.09 | 2.55 | 0.92 | 2.6% | 0.22 | 1.37 | 2.81 | 1.42 | 0.79 | 23.2% | 0.08 | 0.79 | 2.02 | 0.88 | 0.61 |

**Table S8.** Simulated pathogen concentrations in shower/bath water: rate of occurrence, percentiles of concentration when occurring, mean and standard deviation (SD) when occurring. Concentrations are expressed as log_10_ per L; refer to main text for measurement basis.

|  | **5-persons** | | | | | | **100-persons** | | | | | | **1,000-persons** | | | | | |
| --- | --- | --- | --- | --- | --- | --- | --- | --- | --- | --- | --- | --- | --- | --- | --- | --- | --- | --- |
|  | **Occurrence** | **5%** | **50%** | **95%** | **Mean** | **SD** | **Occurrence** | **5%** | **50%** | **95%** | **Mean** | **SD** | **Occurrence** | **5%** | **50%** | **95%** | **Mean** | **SD** |
| Adenoviruses | 0.1% | 3.10 | 5.43 | 7.93 | 5.45 | 1.44 | 2.3% | 1.88 | 4.15 | 6.55 | 4.17 | 1.42 | 20.3% | 1.04 | 3.26 | 5.63 | 3.29 | 1.39 |
| *Campylobacter* | 0.1% | 0.34 | 2.30 | 5.21 | 2.46 | 1.51 | 3.1% | 0.15 | 1.52 | 4.06 | 1.74 | 1.23 | 27.3% | 0.11 | 1.19 | 3.42 | 1.40 | 1.05 |
| *Cryptosporidium* | 0.1% | 0.82 | 2.82 | 5.13 | 2.86 | 1.28 | 1.2% | 0.23 | 1.66 | 3.83 | 1.79 | 1.11 | 11.3% | 0.11 | 1.12 | 3.06 | 1.29 | 0.93 |
| *Giardia* | 0.6% | 0.36 | 2.06 | 4.37 | 2.17 | 1.22 | 11.3% | 0.13 | 1.25 | 3.29 | 1.41 | 0.99 | 69.8% | 0.10 | 0.98 | 2.72 | 1.13 | 0.83 |
| *Norovirus* | 2.8% | 0.61 | 3.22 | 6.99 | 3.48 | 2.01 | 44.8% | 0.30 | 2.77 | 6.00 | 2.90 | 1.81 | 99.7% | 1.23 | 3.90 | 5.96 | 3.80 | 1.40 |
| *Rotavirus* | 0.1% | 3.07 | 5.47 | 7.85 | 5.45 | 1.44 | 2.2% | 1.87 | 4.13 | 6.55 | 4.16 | 1.42 | 19.8% | 1.04 | 3.26 | 5.64 | 3.29 | 1.39 |
| *Salmonella* | 0.1% | 0.87 | 2.97 | 5.20 | 2.99 | 1.30 | 2.6% | 0.28 | 1.81 | 3.97 | 1.92 | 1.13 | 23.2% | 0.13 | 1.24 | 3.19 | 1.39 | 0.96 |

**Table S9.** Simulated pathogen concentrations in laundry water: rate of occurrence, percentiles of concentration when occurring, mean and standard deviation (SD) when occurring. Concentrations are expressed as log_10_ per L; refer to main text for measurement basis.

|  | **5-persons** | | | | | | **100-persons** | | | | | | **1,000-persons** | | | | | |
| --- | --- | --- | --- | --- | --- | --- | --- | --- | --- | --- | --- | --- | --- | --- | --- | --- | --- | --- |
|  | **Occurrence** | **5%** | **50%** | **95%** | **Mean** | **SD** | **Occurrence** | **5%** | **50%** | **95%** | **Mean** | **SD** | **Occurrence** | **5%** | **50%** | **95%** | **Mean** | **SD** |
| Adenoviruses | 0.1% | 0.92 | 3.72 | 7.03 | 3.81 | 1.85 | 2.3% | 0.40 | 2.67 | 5.85 | 2.83 | 1.68 | 20.3% | 0.25 | 2.09 | 5.04 | 2.29 | 1.49 |
| *Campylobacter* | 0.1% | 0.14 | 1.66 | 4.52 | 1.91 | 1.40 | 3.1% | 0.11 | 1.23 | 3.81 | 1.49 | 1.19 | 27.3% | 0.08 | 1.00 | 3.34 | 1.26 | 1.05 |
| *Cryptosporidium* | 0.1% | 0.20 | 1.79 | 4.59 | 2.00 | 1.36 | 1.2% | 0.10 | 1.20 | 3.63 | 1.45 | 1.13 | 11.3% | 0.08 | 0.94 | 3.10 | 1.18 | 0.97 |
| *Giardia* | 0.6% | 0.14 | 1.40 | 4.00 | 1.65 | 1.23 | 11.3% | 0.09 | 1.01 | 3.25 | 1.25 | 1.02 | 69.8% | 0.07 | 0.84 | 2.82 | 1.06 | 0.89 |
| *Norovirus* | 2.8% | 0.25 | 2.48 | 6.27 | 2.76 | 1.89 | 44.8% | 0.20 | 2.10 | 5.41 | 2.35 | 1.64 | 99.7% | 0.47 | 2.69 | 5.39 | 2.77 | 1.49 |
| *Rotavirus* | 0.1% | 0.98 | 3.72 | 6.98 | 3.82 | 1.84 | 2.2% | 0.39 | 2.66 | 5.83 | 2.82 | 1.67 | 19.8% | 0.25 | 2.08 | 5.04 | 2.28 | 1.49 |
| *Salmonella* | 0.1% | 0.20 | 1.85 | 4.55 | 2.05 | 1.37 | 2.6% | 0.11 | 1.25 | 3.71 | 1.49 | 1.14 | 23.2% | 0.09 | 0.99 | 3.19 | 1.23 | 0.99 |

**Table S10.** Simulated pathogen concentrations in combined greywater: rate of occurrence, percentiles of concentration when occurring, mean and standard deviation (SD) when occurring. Concentrations are expressed as log_10_ per L; refer to main text for measurement basis.

|  | **5-persons** | | | | | | **100-persons** | | | | | | **1,000-persons** | | | | | |
| --- | --- | --- | --- | --- | --- | --- | --- | --- | --- | --- | --- | --- | --- | --- | --- | --- | --- | --- |
|  | **Occurrence** | **5%** | **50%** | **95%** | **Mean** | **SD** | **Occurrence** | **5%** | **50%** | **95%** | **Mean** | **SD** | **Occurrence** | **5%** | **50%** | **95%** | **Mean** | **SD** |
| Adenoviruses | 0.1% | 3.67 | 5.53 | 7.58 | 5.57 | 1.19 | 2.3% | 2.36 | 4.24 | 6.34 | 4.28 | 1.21 | 20.3% | 1.42 | 3.32 | 5.41 | 3.35 | 1.21 |
| *Campylobacter* | 0.1% | 0.37 | 2.28 | 4.97 | 2.42 | 1.42 | 3.1% | 0.15 | 1.47 | 3.85 | 1.66 | 1.16 | 27.3% | 0.10 | 1.13 | 3.19 | 1.32 | 0.98 |
| *Cryptosporidium* | 0.1% | 1.25 | 2.84 | 4.87 | 2.91 | 1.08 | 1.2% | 0.29 | 1.60 | 3.57 | 1.72 | 1.01 | 11.3% | 0.10 | 0.99 | 2.77 | 1.15 | 0.85 |
| *Giardia* | 0.6% | 0.49 | 2.06 | 4.09 | 2.15 | 1.09 | 11.3% | 0.13 | 1.13 | 3.01 | 1.29 | 0.91 | 69.8% | 0.08 | 0.84 | 2.48 | 1.00 | 0.76 |
| *Norovirus* | 2.8% | 0.84 | 3.11 | 6.82 | 3.49 | 1.95 | 44.8% | 0.26 | 2.75 | 5.78 | 2.80 | 1.81 | 99.7% | 1.23 | 3.86 | 5.73 | 3.75 | 1.31 |
| *Rotavirus* | 0.1% | 3.71 | 5.56 | 7.62 | 5.58 | 1.20 | 2.2% | 2.36 | 4.22 | 6.35 | 4.27 | 1.21 | 19.8% | 1.42 | 3.32 | 5.41 | 3.36 | 1.21 |
| *Salmonella* | 0.1% | 1.44 | 3.04 | 4.96 | 3.09 | 1.07 | 2.6% | 0.36 | 1.77 | 3.69 | 1.86 | 1.02 | 23.2% | 0.13 | 1.11 | 2.89 | 1.25 | 0.87 |

**Table S11.** Simulated pathogen concentrations in local wastewater from all sources including toilets: rate of occurrence, percentiles of concentration when occurring, mean and standard deviation (SD) when occurring. Concentrations are expressed as log_10_ per L; refer to main text for measurement basis.

|  | **5-persons** | | | | | | **100-persons** | | | | | | **1,000-persons** | | | | | |
| --- | --- | --- | --- | --- | --- | --- | --- | --- | --- | --- | --- | --- | --- | --- | --- | --- | --- | --- |
|  | **Occurrence** | **5%** | **50%** | **95%** | **Mean** | **SD** | **Occurrence** | **5%** | **50%** | **95%** | **Mean** | **SD** | **Occurrence** | **5%** | **50%** | **95%** | **Mean** | **SD** |
| Adenoviruses | 0.1% | 6.16 | 8.20 | 10.20 | 8.20 | 1.23 | 2.3% | 4.92 | 6.93 | 8.92 | 6.92 | 1.22 | 20.3% | 3.97 | 6.01 | 7.97 | 6.00 | 1.21 |
| *Campylobacter* | 0.1% | 2.46 | 4.83 | 7.51 | 4.90 | 1.54 | 3.1% | 1.21 | 3.54 | 6.23 | 3.61 | 1.51 | 27.3% | 0.59 | 2.75 | 5.35 | 2.84 | 1.44 |
| *Cryptosporidium* | 0.1% | 3.78 | 5.58 | 7.38 | 5.57 | 1.09 | 1.2% | 2.44 | 4.23 | 6.07 | 4.24 | 1.10 | 11.3% | 1.51 | 3.28 | 5.11 | 3.29 | 1.09 |
| *Giardia* | 0.6% | 2.80 | 4.73 | 6.65 | 4.73 | 1.16 | 11.3% | 1.57 | 3.48 | 5.38 | 3.48 | 1.15 | 69.8% | 1.00 | 2.92 | 4.65 | 2.89 | 1.10 |
| *Norovirus* | 2.8% | 3.36 | 5.75 | 9.47 | 6.11 | 1.96 | 44.8% | 2.23 | 4.94 | 8.34 | 5.14 | 1.97 | 99.7% | 3.58 | 6.52 | 8.21 | 6.32 | 1.37 |
| *Rotavirus* | 0.1% | 6.22 | 8.17 | 10.12 | 8.19 | 1.20 | 2.2% | 4.91 | 6.92 | 8.91 | 6.92 | 1.21 | 19.8% | 3.98 | 6.01 | 7.98 | 6.00 | 1.22 |
| *Salmonella* | 0.1% | 3.94 | 5.70 | 7.52 | 5.72 | 1.09 | 2.6% | 2.63 | 4.41 | 6.20 | 4.42 | 1.09 | 23.2% | 1.70 | 3.52 | 5.28 | 3.51 | 1.09 |

**References**

Alonso, E., Santos, A. and Riesco, P. (2004) Micro-organism re-growth in wastewater disinfected by UV radiation and ozone: a micro-biological study. Environmental Technology 25(4), 433-441.

Ashbolt, N., Grabow, W. and Snozzi, M. (2001) Water Quality: Guidelines, Standards and Health. Fewtrell, L. and Bartram, J. (eds), pp. 289-316, IWA Publishing, London, UK.

Atmar, R.L., Opekun, A.R., Gilger, M.A., Estes, M.K., Crawford, S.E., Neill, F.H. and Graham, D.Y. (2008) Norwalk virus shedding after experimental human infection. Emerging Infectious Diseases 14(10), 1553-1557.

Barker, S.F. (2014) Risk of Norovirus Gastroenteritis from Consumption of Vegetables Irrigated with Highly Treated Municipal Wastewater-Evaluation of Methods to Estimate Sewage Quality. Risk Analysis 34(5), 803-817.

Barker, S.F., O'Toole, J., Sinclair, M.I., Leder, K., Malawaraarachchi, M. and Hamilton, A.J. (2013) A probabilistic model of norovirus disease burden associated with greywater irrigation of home-produced lettuce in Melbourne, Australia. Water Res 47(3), 1421-1432.

Casanova, L.M., Gerba, C.P. and Karpiscak, M. (2001) Chemical and microbial characterization of household graywater. Journal of Environmental Science and Health, Part A 36(4), 395-401.

Christova-Boal, D., Eden, R.E. and McFarlane, S. (1996) An investigation into greywater reuse for urban residential properties. Desalination 106(1-3), 391-397.

Crockett, C.S. (2007) The role of wastewater treatment in protecting water supplies against emerging pathogens. Water Environment Research, 221-232.

Deere, D., Krogh, M., White, P., Ferguson, C., Davison, A. and Reid, H. (2006) Microbial quality of grey water, Water Futures for South East Water Ltd., Dundas, NSW.

Deoreo, B., Mayer, P., Dziegielewski, B. and Kiefer, J. (2016) Residential End Uses of Water Study Update – Version 2., Water Research Foundation, Denver, CO.

Fane, S., Ashbolt, N. and White, S. (2002) Decentralised urban water reuse: The implications of system scale for cost and pathogen risk. Water Science and Technology 46(6-7), 281-288.

Feachem, R.G., Bradley, D.J., Garelick, H. and Mara, D.D. (1983) Sanitation and disease: health aspects of excreta and wastewater management, John Wiley and Sons, New York.

Friedler, E. (2004) Quality of individual domestic greywater streams and its implication for on-site treatment and reuse possibilities. Environmental Technology 25(9), 997-1008.

Gilboa, Y. and Friedler, E. (2008) UV disinfection of RBC-treated light greywater effluent: Kinetics, survival and regrowth of selected microorganisms. Water Res 42(4-5), 1043-1050.

Havelaar, A., Pot‐Hogeboom, W., Furuse, K., Pot, R. and Hormann, M. (1990) F‐specific RNA bacteriophages and sensitive host strains in faeces and wastewater of human and animal origin. Journal of applied bacteriology 69(1), 30-37.

Jefferson, B., Palmer, A., Jeffrey, P., Stuetz, R. and Judd, S. (2004) Grey water characterisation and its impact on the selectilon and operation of technologies for urban reuse. Water Science and Technology 50(2), 157-164.

Lowe, K.S. (2007) Influent constituent characteristics of the modern waste stream from single sources: Literature review. Water Intelligence Online 6, 9781843397731.

Lowe, K.S., Tucholke, M.B., Tomaras, J.M., Conn, K., Hoppe, C., Drewes, J.E., McCray, J.E. and Munakata-Marr, J. (2010) Influent Constituent Characteristics of the Modern Waste Stream from Single Sources. Water Intelligence Online 9, 9781780403519.

Maimon, A., Tal, A., Friedler, E. and Gross, A., 2010. Safe on-site reuse of greywater for irrigation-a critical review of current guidelines. Environmental Science & Technology, 44(9), 3213-3220.

Mok, H.F., Barker, S.F. and Hamilton, A.J. (2014) A probabilistic quantitative microbial risk assessment model of norovirus disease burden from wastewater irrigation of vegetables in Shepparton, Australia. Water Res 54, 347-362.

Nasser, A.M. (2015) Removal of Cryptosporidium by wastewater treatment processes: A review. Journal of Water and Health, wh2015131.

Nolde, E. (2000) Greywater reuse systems for toilet flushing in multi-storey buildings–over ten years experience in Berlin. Urban water 1(4), 275-284.

NRMMC-EPHC-AHMC (2006) Australian guidelines for water recycling. Managing health and environmental risks. Phase 1. National water quality management strategy 21.

Ottoson, J. and Stenström, T.A. (2003) Faecal contamination of greywater and associated microbial risks. Water Res 37(3), 645-655.

Pouillot, R., Van Doren, J.M., Woods, J., Plante, D., Smith, M., Goblick, G., Roberts, C., Locas, A., Hajen, W. and Stobo, J. (2015) Reduction of Norovirus and Male-Specific Coliphage Concentrations in Wastewater Treatment Plants: a meta-analysis. Applied and Environmental Microbiology, AEM. 00509-00515.

R Core Team (2015) R: A language and environment for statistical computing, R Foundation for Statistical Computing, Vienna, Austria.

Rose, J.B., Nowlin, H., Farrah, S.R., Harwood, V.J., Lukasik, J., Pepe Menendez, P. and Scott, T.M. (2004) Reduction of pathogens, indicator bacteria, and alternative indicators by wastewater treatment and reclamation processes, Water Environment Research Foundation, Alexandria, VA.

Rose, J.B., Sun, G.S., Gerba, C.P. and Sinclair, N.A. (1991) Microbial Quality and Persistence of Enteric Pathogens in Graywater from Various Household Sources. Water Res 25(1), 37-42.

Santos, C., Matos, C. and Taveira-Pinto, F. (2014) A comparative study of greywater from domestic and public buildings. Water Science and Technology-Water Supply 14(1), 135-141.

Soller, J.A., Eftim, S., Warren, I. and Nappier, S. (2016) Evaluation of microbiological risks associated with direct potable reuse. Microbial Risk Analysis.

Stampi, S., Varoli, O., Zanetti, F. and De Luca, G. (1993) Arcobacter cryaerophilus and thermophilic campylobacters in a sewage treatment plant in Italy: two secondary treatments compared. Epidemiology and Infection 110(03), 633-639.

Winward, G.P., Avery, L.M., Frazer-Williams, R., Pidou, M., Jeffrey, P., Stephenson, T. and Jefferson, B. (2008) A study of the microbial quality of grey water and an evaluation of treatment technologies for reuse. Ecological Engineering 32(2), 187-197.
